# Comparison of vector velocity imaging and three-dimensional speckle tracking echocardiography for assessment of left ventricular longitudinal strain in hypertrophic cardiomyopathy

**DOI:** 10.21542/gcsp.2019.6

**Published:** 2019-03-31

**Authors:** Hala Mahfouz Badran, Naglaa Faheem, Mahmood Soliman, Mohamed Hamdy, Magdi Yacoub

**Affiliations:** 1 Cardiology Department, Menoufiya University, Egypt; 2 The BAHCM National Program, Egypt; 3 Aswan Heart Center, Aswan, Egypt; 4 Imperial College, London, UK

## Abstract

**Background:** Vector velocity imaging (VVI) is a two-dimensional wall motion tracking method that can measure cardiac mechanics in hypertrophic cardiomyopathy (HCM). 3D-speckle tracking echocardiography (3D-STE) has been proven to be superior to conventional measures in assessment of LV function. The aim of this study was to compare the two technologies in the assessment of LV longitudinal strain (LS) in HCM patients.

**Methods:** A total of 50 patients with HCM were investigated using VVI and 3D-STE in same setting. 3D-STE allows obtaining longitudinal, circumferential, radial and area strains (AS). Values of longitudinal strain (LS) and AS by 3D-STE were compared to VVI- derived analyses. Thereafter, VVI-LS values were correlated with LV phenotype. Last, the variability of VVI versus 3DE strain measurements as well as recorded time of analysis was assessed.

**Results:** The absolute value of 3D-STE LS and AS is significantly higher than VVI-LS (*P* < 0.0001). VVI provided complete longitudinal LV strain information, similar to 3D-STE. There is excellent agreement between the two technologies-derived values, however, a greater number of segments could be analyzed using VVI (94.7%), compared with 3DE (62.1%). Despite VVI being more time consuming, VVI-LS is more correlated to LV mass index, mitral regurgitation severity and functional class when compared with 3D-STE LS and AS.

**Conclusions:** VVI is a feasible modality for assessing LV longitudinal strain. Although VVI agreed well with 3D-STE for most of regional and global LS, a better correlation was found between VVI-LS and HCM phenotype. It is hypothesized that this discrepancy originates from the inferior imaging quality using 3D tracking algorithms.

## Introduction

LV ejection fraction (LVEF) is the most popular index of myocardial systolic function. However, several studies suggested that LVEF is a poor indicator for detecting early myocardial dysfunction^1–3^. Hypertrophic cardiomyopathy (HCM) is the best working example – a relatively common inherited cardiomyopathy that is occasionally challenging, especially with recent evidence of systolic functional abnormalities despite normal LVEF^1–3^.

The assessment of myocardial mechanics using strain imaging not only describes function, but also provides valuable information regarding prognosis in a variety of disease processes, such as coronary artery disease^4^ and idiopathic dilated cardiomyopathy^5^.

Doppler tissue imaging–derived strain imaging has shown feasibility for measuring myocardial function, however, it can only quantify the axial component of motion, and so is angle-dependent^6^. Recently, a new feature-tracking echocardiographic method using velocity vector imaging (VVI) is applied through a combination of mitral annulus motion, tissue-blood border detection, and speckle tracking. It has previously been validated against crystal sonomicrometry^7^, providing an angle-independent principle of myocardial deformation^8,9^. However, two-dimensional wall motion–tracking echocardiography (2DE) is limited because acquisition and analysis are time consuming.

The recently-developed three-dimensional speckle-tracking echocardiography (3D-STE) is a promising tool to precisely quantify LV strain using complete 3D pyramidal data sets. Preliminary studies show that 3D-STE may overcome the limitations of Doppler-based strain or 2D-based speckle-tracking strain images^10,11^.

The primary objectives of this study were to compare VVI longitudinal strain measurements derived from 2DE against those derived from 3D-STE, performed in patients with HCM who underwent both studies on the same day, and to evaluate the usefulness of each method to reflect the underlying pathology in such complex disease.

## Methods

### Study population and protocol

This was a single-centre observational study. We enrolled 80 consecutive HCM patients between September 2012 and November 2013. Patients were examined in a single centre (Yacoub Research Unite, Menoufiya University, Egypt). The diagnosis of HCM was based on conventional echocardiographic demonstration of a non-dilated, hypertrophic LV (≥15 mm) in the absence of other cardiac or systemic diseases capable of producing the magnitude of hypertrophy evident ^12^.

The exclusion criteria included patients with LVEF <50%, prior myectomy or alcohol septal ablation, ICD, diabetes mellitus, hypertension, evidence of coronary artery disease, atrial fibrillation, lung disease and patients with suboptimal image quality unsuitable for strain measurements^9^.

Enrolled patients were subjected to standard echocardiography, 2D- VVI and 3D-STE in the same setting. Written informed consent was obtained from all subjects and the study was approved by the institution’s research ethics committees.

### Echocardiography

All subjects were examined in the left lateral decubitus position using an Esaote Mylab Gold 30 ultrasound system (Esaote S.p.A, Florence, Italy) equipped with a 5 MHz phased-array transducer. Standard techniques were used to obtain M-mode, LV wall thickness and dimensions, 2D, and Doppler measurement in accordance with the American Society of Echocardiography guidelines^13^. The LVEF was measured using Simpson’s biplane method. The medial mitral annular early diastolic velocity (E_a_) – assessed by pulsed-wave tissue Doppler imaging – was recorded to derive E/ E_a_.

### VVI longitudinal strain analysis

VVI is an angle-independent feature-tracking method that incorporates speckle-tracking and endocardial and epicardial contour tracking. VVI quantifies myocardial motion from B-mode clips by automatically tracking user-defined endocardial and epicardial contours to define the inward and outward myocardial motion. On the basis of the motion of the tracked points between the frames and the knowledge of the time interval between frames, 2D tissue velocity is computed. Strain and strain rate are computed by the change in the relative distance between localized tracked trace points, combined with the difference in the relative displacement of the tissue motion behind the tracked points.

Border tracking of the LV was manually traced from the digitized 2D video clips recorded during breath holding and with good quality ECG signal which was acquired and stored during three cardiac cycles, in digital format for subsequent offline analysis using XStrain™ software with a frame rate between 40–80 fps. Scanning was performed from the apex to acquire best apical 2, 3, and 4 chamber views. Endocardial borders were manually traced on the end-diastole frame of each single loop to initialize the automatic tracking algorithms. Manual adjustment of each border independently of the other could be performed before analysis and processing. Longitudinal strain parameters were recorded after visual confirmation of best wall motion tracking (by operator subjective visual assessment). Segments with poor tracking were excluded from further analysis. Global values of LS were obtained by averaging the segmental strain values (six segments in each of the three apical views, for a total of 18 segments).

### 3D speckle-tracking echocardiography analysis

For 3DE, a fully sampled matrix-array transducer with almost 3,000 active elements was used (4V-D; *Vivid* 9 (GE Ultrasound, Horten, Norway)) ultrasound system. The 3DE data acquisition was obtained in an adjustable volume divided into six sub-volumes. The acquisition of sub-volumes was steered electronically with the transducer kept in a stable position at the LV apex. The acquisition was triggered to the R wave of the ECG on consecutive heartbeats and obtained in an end-expiratory breath-hold long-lasting.

**Figure 1.**
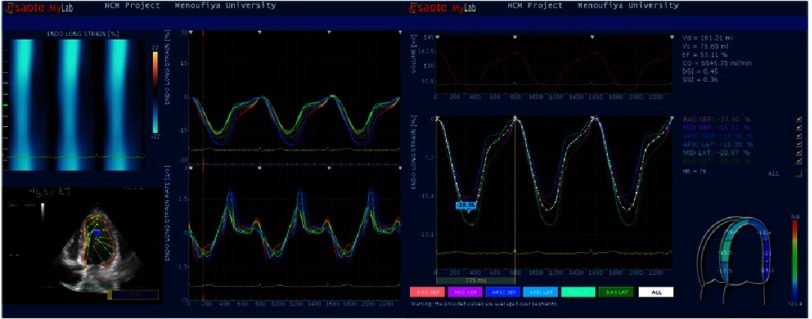
Strain curves of septal and lateral wall segments in HCM patient. Mean peak strain from LV wall segments is 18.36%.

**Figure 2.**
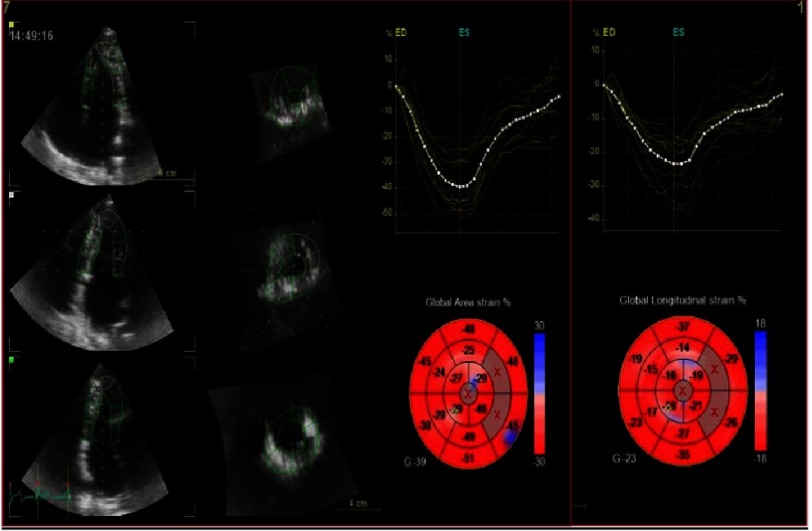
3D-STE offline analysis. Semi-automated endocardial border identification and tracking. The analysis of LV strain parameters can be calculated and displayed on bull’s eye. Global longitudinal strain (GLS) is 23% and global area strain (GAS) is 39%.

Data acquisition was conducted in several stages^10,11^. First, alignment with presentation of all apical views, as well as the transversal plane, was performed. Orientation was performed automatically. Second, for end-diastolic volume, the operator placed, in end-diastole, one point in the middle of the mitral annulus plane and a second point at the LV apex. The software generated an end diastolic endocardial border tracing. Papillary muscles were included within the LV cavity. Adjustments of the automatic tracing were performed manually by the operator to reposition it closer to the endocardial border. Third, for end-systolic volume, the same process was repeated in end-systole. The analysis enabled the acquisition of LV volumes and LV EF. Fourth, for the volume waveform, the correct alignment of the contours with the endocardium during the cardiac cycle was controlled. Fifth, a second semi-automated delineation was made in relation to the epicardium to delineate the region of interest for strain analysis. Last, after handling by the software, the final 3D-STE analysis allowed the measurement of 3D global longitudinal strain (GLS), global circumferential strain, global radial strain and global area strain (GAS), were computed at end-systole (Figures 1, 2).

We preferred to analyze 3D GLS and GAS as it reflects both longitudinal and circumferential strain vectors and compare it to VVI derived GLS.

The duration of 3D-STE image acquisition and analysis to obtain strain data, were calculated and compared with that for VVI analysis.

#### Reproducibility analysis

Intra- and inter-observer variability was evaluated in a random sample of 20 subjects selected by a random number table. To test intra-observer variability, the same primary observer analyzed the data twice at least 2 weeks apart. During these repeated analyses, the observer was blinded to the results of all prior measurements. To test inter-observer variability, a second experienced observer, who was blinded to the first observer’s measurements, analyzed the same data. Intra- and inter-observer variability was calculated as the ratio of the absolute difference between two measurements over the mean of those measurements and evaluated by means of intra-class correlation coefficients (ICCs).

## Results

A total of 80 subjects were evaluated for inclusion in the study. Of those, 30 were subsequently excluded: 17 subjects because of low frame rates as determined by the software system, and 13 because of poor 3D images. Thus, the final study group consisted of 50 patients (mean age, 41.5 ± 16 years).

The population comprised 32 (64 %) men with LV EF% 70 ± 11 by 2D imaging using Simpson’s methods.

41 (82%) of the patients showing asymmetrical type of LVH with maximal wall thickness (MWT) of 27.8 ± 7.7.

40 (80%) of the study cohort had no or mild MR, and 13 (26%) showed SAM criteria. Clinical and echocardiographic characteristics of the study population are depicted in Table 1.

### Comparison of segmental and GLS between VVI and 3DE analyses

Of the 2,778 potential segments available for longitudinal strain 2,630 (94.7%) were analyzable using VVI and 1700 (61.2%) using 3D-STE. The absolute values of segmental strain of VVI (−8.74 ± 6.7%) were significantly lower compared to 3DE values (−13.8 ± 5.7%), (*P* < 0.0001), (Table 2).

A significant decrease in the septal segmental strain in VVI versus 3D-STE (*P* < 0.0001), coupled with decreased mid- and apical-inferior segmental strain values resulted in a significant reduction of GLS in VVI compared to 3DE analysis. Overall the mean differences in GLS for the two imaging modalities were 4.1  ± 2.3% and between 3DE-GLS & GAS was 12.6 ±4.5%. Both 3D regional and GAS showed significantly higher value compared to GLS measured by VVI (*P* < 0.00001).

**Figure 3.**
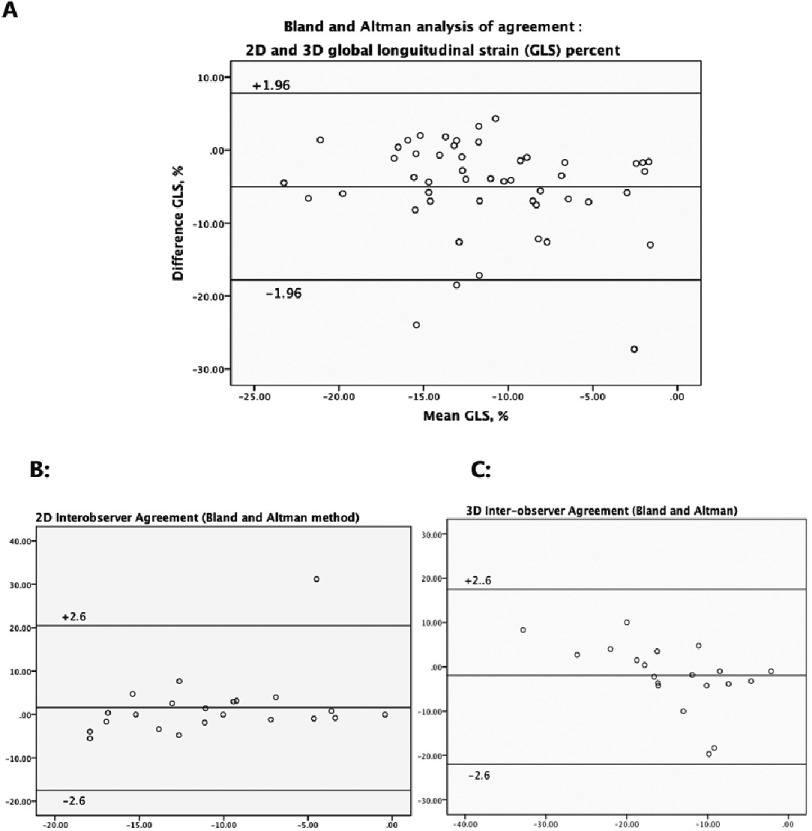
Bland-Altman analysis. A, Agreement of longitudinal strain between 3D-STE and 2D-VVI; B, 3D-STE inter-observer agreement; C, VVI inter-observer agreement of global longitudinal strain.

There was excellent agreement for regional and global LV longitudinal strain between VVI and 3D-STE strain at *r* = 0.462 (y = 1.33x + 15). With a wide dispersion by linear regression, with Bland-Altman analysis, 3D measurement of GLS can be larger by 17.7 or smaller by 7.7 - in absolute values - as compared to 2D measurements (Table 3). The highest correlation was noted in the lateral wall *r* = 0.699, and the least in the septum *r* = 0.506 (inferior wall *r* = 0.593, anterior wall *r* = 0.613). While a lower agreement was observed between VVI GLS and 3D GAS (*r* = 0.398, *P* < .007) (Figure 3).

#### Base to apex strain gradient using VVI versus 3D-STE

In HCM no significant difference in the strain gradient from base to apex using VVI nor 3D-STE was observed, as illustrated in Table 3.

#### Assessment of agreement and repeatability between VVI and 3D-STE

The total study time was significantly shorter with 3DE than with VVI, including the manual initialization (4.3 ± 1.2 versus 6.1 ± 1.4 min; *P* < 0.01), and this was the case for both acquisition time and analysis time (*P* < 0.001). For VVI GLS, the intra-observer and inter-observer coefficients of variation (CV) were 2.3 ± 0.22 and 2.6 ± 0.25% respectively. Interclass correlation coefficients (ICC) ranged from 0.85 to 0.94. For 3D GLS the intra-observer and inter-observer CV was 5.7 ± 4.3 and 6.2 ± 5.5% respectively, and ICC ranged from 0.79 to 0.87. While for 3D GAS the intra-observer and inter-observer CV was 5.5 ± 4.2 and 7.9 ± 5.3% respectively, and ICC ranged from 0.82 to 0.90 (Table 4) (Figures 3A, 3B, 3C).

**Table 1:** Basal characteristics of study population.

	HCM (*n* = 50)
Age (years)	41.5 ± 16
Male:	32 (64%)
BSA (Kg/m2)	1.8 ± 0.28
HR (b/min)	73 ± 17
SBP (mmHg)	126 ± 21
DBP (mmHg)	79 ± 14
Functional class	2.14 ± 0.5
ESD (mm)	22.58 ± 6.09
EDD (mm)	36.79 ± 7.16
FS%	39 ± 9
EF%	70 ± 11
MWT (mm)	27.8 ± 7.7
SPT (mm)	26.2 ± 6.8
LVPW (mm)	14.9 ± 4.4
S/PW Ratio	1.92 ± 0.84
Type of LVH	
Concentric:	9(18%)
Asymmetric:	41(82%)
LVM(gm)	440 ± 174
LVMI (gm/m ^2^)	246.6 ± 92.8
LVOTG (mmHg)	23.8 ± 30.6
MR	
No/Mild:	40 (80%)
Moderate:	7 (14%)
Severe:	3 (6%)
SAM	13 (26%)
Mitral E(m/s)	.84 ± .31
Mitral A(m/s)	1.78 ±
mitral E/A	1.4 ± .68
DT(ms)	202 ± 65
PAP (mmHg)	24 ± 7

**Notes.**

BSA body surface area SBP systolic blood pressure DBP diastolic blood pressure LVH left ventricular hypertrophy SAM systolic anterior motion LVOTG LV outflow tract, gradient ESD end-systolic diameter EDD end-diastolic diameter EF ejection fraction MWT maximal wall thickness PWT posterior wall thickness SPT septal thickness LVMI left ventricular mass index E early mitral inflow velocity A atrial mitral inflow velocity DT deceleration time PAP pulmonary artery pressure E_a_ mitral annulus early diastolic velocity

**Table 2:** Comparison of 2D versus 3D LV strain measurements in HCM.

	VVI-LS %	3D-LS %	3D-AS%
Basal septum	−9.44 ± 7.21	−12.25 ± 5.9^b^	−25.6 ± 9^d^
Mid septum	−9.78 ± 7.07	−15.2 ± 6.81^c^	−25.4 ± 8.8^d^
Apical septum	−12.9 ± 8.49	−18.3 ± 10.07^b^	−27.7 ± 13^d^
**Mean septum**	**−10.81 ± 6.61**	**−15.8 ± 7.11^c^**	**−27 ± 8.9^d^**
Basal lateral	−13.1 ± 8.79	−19.1 ± 9.8^b^	−30.2 ± 12^d^
Mid lateral	−9.14 ± 6.99	−13.3 ± 7.91^c^	−27 ± 10.8^d^
Apical lateral	−9.58 ± 6.451	−11.2 ± 7.24	−25.7 ± 10.5^d^
**Mean lateral**	**−10.54 ± 6.71**	**−13.6 ± 7.07^c^**	**−27.5 ± 8.4^d^**
Basal anteroseptum	−10.2 ± 5.11	−13.04 ± 5.9^b^	−24.1 ± 6^d^
Mid anteroseptum	−9.89 ± 7.07	−14.1 ± 5.7^c^	−23.9 ± 9.5^d^
Basal Posterior	−11.4 ± 6.2	−10.8 ± 6.25	−26.3 ± 5.5^d^
Mid posterior	−9.9 ± 7.8	−13.2 ± 6.7^a^	−22.8 ± 8.3^d^
Basal anterior	−13.60 ± 9.68	−15.8 ± 9.23	−30 ± 11.2^d^
Mid anterior	−11.25 ± 6.83	−11.9 ± 8.24	−24.5 ± 10^d^
Apical anterior	−10.48 ± 7.12	−9.75 ± 8.57	−24 ± 9.9^d^
**Mean anterior**	**−11.78 ± 6.61**	**−12.1 ± 8.41**	**−26.7 ± 8.2^d^**
Basal inferior	−11.40 ± 7.23	−11.8 ± 7.35	−27.7 ± 8.5^d^
Mid inferior	−9.87 ± 6.73	−13.4 ± 6.69^a^	−23.9 ± 9.3^d^
Apical inferior	−11.15 ± 8.83	−14.8 ± 8.15^a^	−26.5 ± 12.5^d^
**Mean inferior**	**−10.8 ± 6.76**	**−13.6 ± 6.33^b^**	**−26.3 ± 8.3^d^**
**GLOBAL STRAIN %**	**−8.74 ± 6.74**	**−13.8 ± 5.73^c^**	**−26.4 ± 6.8^d^**

**Notes.**

^a^ *P* < 0.01, ^b^ *P* < 0.001, ^c^ *P* < 0.0001, ^d^ *P* < 0.00001 versus VVI.

VVI vector velocity imaging LS longitudinal strain AS area strain

**Table 3:** Base to apex gradient of longitudinal strain.

	VVI	3D-STE
Base (%)	11.9 ± 8.3	14.7 ± 8.7
Mid (%)	10.0 ± 6.9	13.5 ± 7.4
Apex (%)	11.0 ± 7.8	13.5 ± 9.0

**Notes.**

VVI two-dimensional vector velocity imaging 3D-STE three-dimensional speckle tracking echocardiography

**Table 4:** Comparison between VVI and 3D longitudinal strain.

Variable	2D-VVI	3D-STE	*P*-value
Analyzed segments (%)	94.7%	61.2%	<0.001
Acquisition time (min)	3.2 ± 1.3	1.6 ± 0.8	<0.01
Analysis time (min)	3.3 ± 1.2	2.9 ± 0.7	<0.01
Total time (min)	6.1 ± 1.4	4.3 ± 1.2	<0.01
Intra-observer variability (%)	2.3 ± 0.22	5.7 ± 4.3	<0.001
Inter-class correlation (%)	92%	84%	<0.01
Inter-observer variability (%)	2.6 ± 0.25	6.2 ± 5.5	<0.002

**Notes.**

2D-VVI two-dimensional vector velocity imaging 3D-STE three-dimensional speckle tracking echocardiography

#### Relation of LV phenotype to VVI and 3DE longitudinal strain

**Figure 4.**
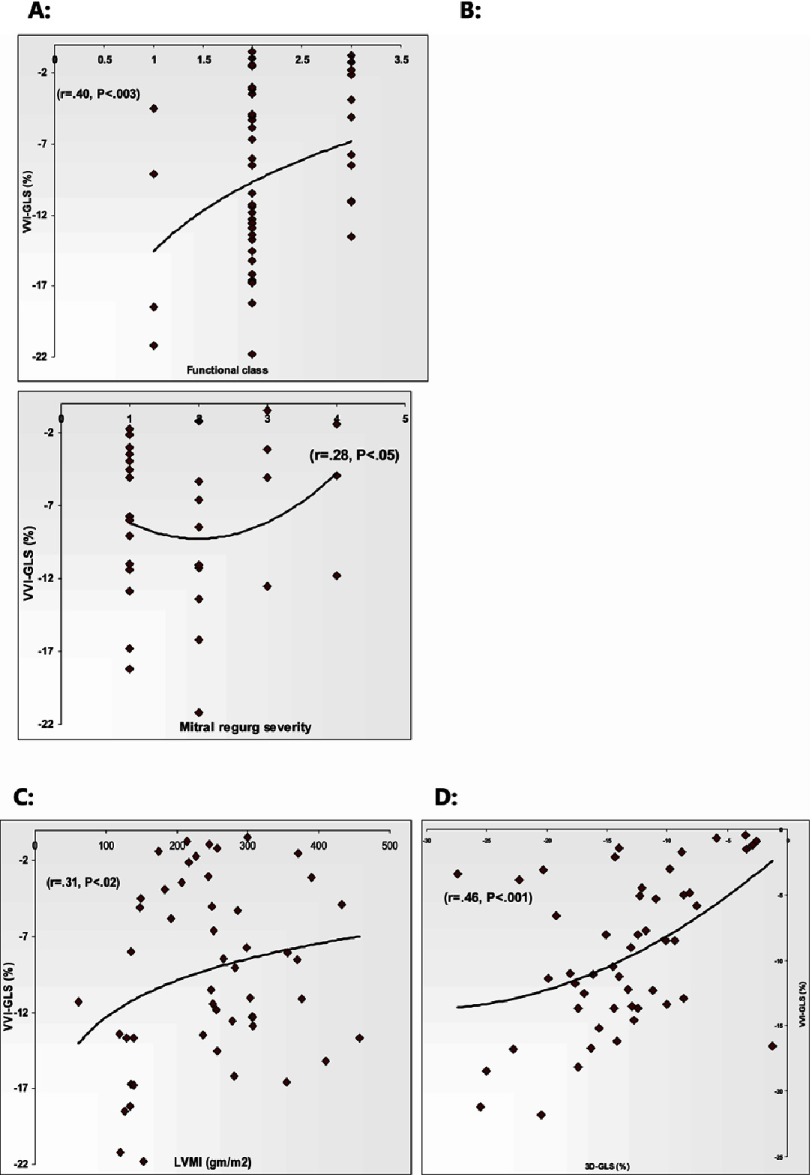
Correlation of VVI- GLS to HCM phenotype. A, Functional class; B, Mitral regurge severity; C, Left ventricular mass index; D, 3D-GLS.

As illustrated in Table 5 (and Figure 4) the only correlation with functional class was obtained with VVI-GLS, but notably not with 3D-STE GLS measurements. Both VVI-GLS and 3DE-GAS showed an inverse relationship with the severity of mitral regurgitation (*P* < 0.04, 0.02) respectively. There was no significant correlation between VVI-GLS, 3D-GLS, or GAS and EF% or MWT (P = NS) –whereas, when taking LV septal thickness separately, it showed inverse correlation to regional septal strain as measured by VVI (*r* = 0.322, *P* < 0.01), but not with 3DE (*r* = 0.201 for GLS and *r* = 0.241 for GAS, p = NS) (Figure 3). Furthermore there was significant inverse relationship between VVI-GLS, 3D-GLS and GAS with LVMI (*P* < 0.02, 0.02, 0.01) respectively.

**Table 5:** Relation of VVI-GLS & 3D-GLS & GAS to LV phenotype in HCM.

	Functional class		Mitral regurgitation		EF		MWT		LVMI	
	r	P	r	P	r	P	r	P	r	P
VVI-GLS	0.400	0.003	−0.277	0.045	−0.066	0.650	0.216	0.07	−0.309	0.021
3D-GLS	0.248	0.101	0.245	0.102	−0.005	0.975	0.197	0.373	−0.306	0.023
GAS	0.067	0.523	−0.306	0.023	−0.081	0.576	0.170	0.446	−0.345	0.012

**Notes.**

EF ejection fraction MWT maximal wall thickness LVMI left ventricular mass index VVI vector velocity imaging GLS global longitudinal strain 3D three dimensional GAS global area strain

## Discussion

In the present study, we have demonstrated that the application of VVI to measure LV longitudinal strain is both feasible and reproducible, providing excellent agreement with values derived from three-dimensional echocardiography in patients with HCM. While 3DE is less time consuming, VVI showed superior correlation to phenotype – however, the two imaging techniques have comparable intra-observer and inter-observer variability.

Hypertrophic cardiomyopathy (HCM) is one of the most common inherited cardiac disorders, characterized by diastolic dysfunction as an early clinical feature in patients with both positive and negative phenotype^3^. With the development of recent echocardiograhgic technologies, such as strain imaging and 3D echocardiography, concealed systolic dysfunction even in asymptomatic patients have been identified. 2D strain identified a subclinical systolic dysfunction in HCM and it became able to differentiate HCM from hypertensive cardiomyopathy and athlete’s heart^14^.

2D strain analysis using STE measures myocardial deformation by means of frame-by-frame tracking and motion analysis of speckles within B-mode images. Validation studies with tagged CMR imaging and sonomicrometry in the different populations^15,16^, have provided evidence that 2D-STE is a reliable method to determine ventricular myocardial function. However, 2D-STE is limited by a number of factors. First, myocardial deformation measured is affected by loss of speckles due to motion outside the imaging plane^17^. Second, 2D-STE has limited reproducibility, likely because of variability in the choice of image planes and a lack of standardization in image analysis^18^. Finally, the analysis is cumbersome, and there is a need for a total of six planes for complete analysis, which is a major limitation for automation and potential clinical use.

More recently, the advent of 3D-STE has the potential to overcome the limitations of 2D-STE for the assessment of LV global and regional systolic function^19,20^. This method tracks the motion of speckles within the scan volume, allowing more complete and accurate assessment of myocardial deformation in all three spatial dimensions^21^ by avoiding the loss of speckles because of out-of-plane motion. It has been validated for the quantification of LV volumes^22^ and LV wall motion in ischemic heart disease^23^ in adults. Nonetheless, data are scarce and incomplete for the other myocardial disease and in pediatric population.

Thus, the current study investigated the establishment of feasibility, reproducibility, of VVI as new feature tracking technology and compared it to 3D-STE in complex disease like HCM, as a prerequisite for evaluation of LV deformation.

VVI is a novel echocardiographic imaging technique built on routine 2D- gray scale images. It is based on myocardial feature tracking, which incorporates speckle, mitral annular motion and endocardial border tracking^9^. Tracking is performed by following reference points, which guide the detection of adjacent points, and snake contours are used to track the whole length of the endocardial border^18^. This may potentially yield results that are more robust than those obtained by algorithms that only track speckle in echocardiography. VVI assesses innermost myocardial function adjacent to the endocardial border. This makes the tracking process more precise and allows myocardial strains, strain rates, and velocities to be quantified, in an angle-independent principle.

VVI can be used to study cardiac mechanics and quantify global and regional cardiac function with abundant parameters. It quantifies circumferential and longitudinal myocardial deformation, and the latter is important particularly in early detection of dysfunction at the subclinical level. VVI is faster than conventional 2D-STE, and obtaining each patient’s parameters may take less time than in routine STE study^8,9,12^. In the present study the total time of acquisition in the three long axis views and analysis time took around 4 to 7 minutes, which was somewhat longer than 3DE (3 to 5 minutes).

In the present study, successful measurements of longitudinal strain were obtained in ∼95% of cases, which was significantly greater than the number analyzed using 3DE (62%). Actually, the new 3D wall motion–tracking system works well with good-quality echocardiographic images. 3D-STE is limited by significant dependence on the quality of the ultrasound window and acquisition, particularly in relation to endocardial boundary delineation. In addition, the ability to track anatomic details frame by frame is affected by the random image noise and the relatively low temporal resolution^19^ . In fact, 13 patients were excluded from the current study because of poor 3D acoustic windows, while they were acceptably analyzed using VVI. The feasibility of VVI has been previously examined in the contexts of response to cardiac resynchronization therapy^24^, heart function^25^, and left atrial function^26^. In addition, the accuracy of VVI for assessing regional myocardial function using strains has been confirmed by comparative tissue velocity imaging^9^ and sonomicrometry^7^.

Our study showed a modest correlation between GLS by 3DE and VVI analysis (*r* = 0.46). Also, GAS was shown to better correlate with LV phenotype than 3DE-GLS. The current data show comparable intermodality agreement with previous studies comparing VVI derived strain values with those obtained with magnetic resonance imaging tagging (tMRI)^27^ and comparing 2D-STE derived strain values with those obtained with (tMRI)^28^.

The reproducibility of VVI longitudinal strain marker appeared to be suitable for clinical use, being even superior to that of 3DE imaging for longitudinal mechanics analyses. 3D-STE analysis is highly dependent on image quality, and its low frame rate possibly may lead to miscorrelation between frames and affect the accuracy of strain data. 3DE evaluation was shown to be more robust than the data obtained using VVI imaging, as there is a need for three planes for complete analysis, which represents a negative aspect for timely practical application. However, we consider VVI a reasonable modality for precise assessment of LV longitudinal mechanics

### Study limitations

Several important limitations can be considered. The present validation study was performed only in patients with HCM; therefore, it is unknown whether the results of the present study can be extrapolated to other cardiac pathologies. The presence of LV hypertrophy provides superior conditions for feature-tracking, and it is yet to be determined whether correlations are as favorable in the presence of normal wall thickness. Although VVI and 3DE scans were performed <24 hours apart, it is possible that some of the differences demonstrated in strain parameters between the two imaging modalities may be explained by alteration in the underlying hemodynamic state (particularly dynamic LV outflow tract obstruction). We did not analyze circumferential and radial strains because obtaining uniformity of the tomographic plane is easier to achieve with long-axis (represent 75% of cardiac function) than short-axis imaging views, so the differences between measurements may be affected by imaging planes rather than by the technology applied.

## Conclusion

VVI is a feasible modality for assessing LV longitudinal strain. It agreed well with 3D-STE for most of regional and global LS. It provides more complete and reproducible tool in assessment of global LV long axis function. While VVI revealed less robust analysis, a better correlation was found between VVI-LS and HCM phenotype. It is hypothesized that this discrepancy originates from the inferior imaging quality using 3D tracking algorithms. Further research should be performed to assess VVI as a potential clinical bedside tool for quantifying LV deformation in different myocardial diseases.
